# Temporal and molecular dynamics of human metastatic breast carcinoma cell adhesive interactions with human bone marrow endothelium analyzed by single-cell force spectroscopy

**DOI:** 10.1371/journal.pone.0204418

**Published:** 2018-09-20

**Authors:** Leike Xie, Zhe Sun, Zhongkui Hong, Nicola J. Brown, Olga V. Glinskii, Kate Rittenhouse-Olson, Gerald A. Meininger, Vladislav V. Glinsky

**Affiliations:** 1 Dalton Cardiovascular Research Center, University of Missouri, Columbia, Missouri, United States of America; 2 Department of Pathology and Anatomical Sciences, School of Medicine, University of Missouri, Columbia, Missouri, United States of America; 3 Microcirculation Research Group, Department of Oncology, School of Medicine, University of Sheffield, Sheffield, United Kingdom; 4 Department of Medical Pharmacology and Physiology, School of Medicine, University of Missouri, Columbia, Missouri, United States of America; 5 Research Service, Harry S. Truman Memorial Veterans Hospital, Columbia, Missouri, United States of America; 6 Department of Biotechnical & Clinical Laboratory Sciences, University at Buffalo, Buffalo, New York, United States of America; 7 For-Robin, Inc, Buffalo, New York, United States of America; LAAS-CNRS, FRANCE

## Abstract

Bone is a common site of metastasis for breast cancer and the mechanisms of metastasis are not fully elucidated. The purpose of our study was to characterize temporal and molecular dynamics of adhesive interactions between human breast cancer cells (HBCC) and human bone marrow endothelium (HBME) with piconewton resolution using atomic force microscopy (AFM). In adhesion experiments, a single breast cancer cell, MDA-MB-231 (MB231) or MDA-MB-435 (MB435) was attached to the AFM cantilever and brought into contact with a confluent HBME monolayer for different time periods (0.5 to 300 sec). The forces required to rupture individual molecular interactions and completely separate interacting cells were analyzed as measures of cell-cell adhesion. Adhesive interactions between HBME and either MB231 or MB435 cells increased progressively as cell-cell contact time was prolonged from 0.5 to 300 sec due to the time-dependent increase in the number and frequency of individual adhesive events, as well as to the involvement of stronger ligand-receptor interactions over time. Studies of the individual molecule involvement revealed that Thomsen-Friedenreich antigen (TF-Ag), galectin-3, integrin-β1, and integrin-α3 are all contributing to HBCC/HBME adhesion to various degrees in a temporally defined fashion. In conclusion, cell-cell contact time enhances adhesion of HBCC to HBME and the adhesion is mediated, in part, by TF-Ag, galectin-3, integrin-α3, and integrin-β1.

## Introduction

Bone is one of the major sites of breast cancer metastasis. Seventy percent of patients suffering from advanced breast cancer develop bone metastasis [1]. There are currently no effective therapies available to prevent or treat breast cancer metastasis to the bone [2–3]. Metastasis is a very complex process, which begins with successful escape of tumor cells from the primary site, penetration into and survival within the circulation, arrest and extravasation at remote sites, and culminates with invasion of target tissue and proliferation of metastatic lesions [4–7]. Adherence of a circulating tumor cell to vascular endothelial cells is an essential process for extravasation from the vasculature [7–10]. The mechanisms regulating metastatic tumor cell interactions with endothelial cells in distant organs are incompletely understood, despite numerous biological and clinical studies investigating the pathogenesis of cancer metastasis [11–18]. A better understanding of the characteristics of interactions between tumor cells and endothelial cells, and the molecular mechanisms underpinning these interactions, continues to be a key for developing approaches to reduce the incidence of metastasis and for the development of new therapeutic and diagnostic strategies.

Several molecules such as Thomsen-Friedenreich antigen (TF-Ag), galectin-3 (Gal-3) and different integrins are involved in adhesive interactions between cancer cells and endothelial cells [11,13,19]. TF-Ag is a disaccharide galactose β1-3N-acetyl galactosamine conjugated to proteins by an O-serine or O-threonine linkage and is expressed on the cell surface of most human carcinomas, including breast cancer cells [20–22]. This well-defined carbohydrate antigen plays a leading role in the initial adhesion of breast cancer cells to vascular endothelium by specifically interacting with endothelial Gal-3 [11]. Gal-3 is a carbohydrate-binding protein expressed in most human cells, including tumor and endothelial cells [23–25]. However, only the Gal-3 expressed in endothelium, rather than in tumor cells, mediates tumor/endothelial cell adhesion via interactions with cancer associated TF-Ag [13]. Gal-3 is commonly present in endothelial cytoplasm and can translocate to the cell surface upon endothelial activation by TF-Ag expressing cancer cells [11,13,21,26]. Integrins are transmembrane adhesion proteins that form heterodimers of alpha and beta subtypes and are expressed in both tumor and endothelial cells [19,27–28]. It has been shown that integrin α3β1 (α3β1) expressed in cancer cells not only promotes cancer invasion [29–31], but also mediates cancer cell adhesion to vascular endothelium in metastasis [32]. In addition, α3β1 expressed in endothelial cells is proposed to play an important role in stabilizing TF-Ag/Gal-3 mediated tumor-endothelial adhesion [13].

Atomic force microscopy (AFM) is a highly sensitive force measuring technique that has been proven to be useful for investigating the adhesive interactions of living cells under physiological conditions [33–34]. Quantitation of adhesion forces between cancer and endothelial cells is obtained using AFM single-cell force spectroscopy in which a single cancer cell is attached to the tip of a cantilever and brought into contact with an endothelial monolayer that grows on a substrate. The ligand-receptor rupture events and total adhesion forces are calculated from the cantilever deflections monitored during cantilever retraction [34–35]. In the present study, we used AFM to characterize adhesive interactions between individual human breast cancer cells and a human bone marrow endothelial (HBME) cell monolayer and identify molecules that are involved in the adhesion of breast cancer cells to HBME with functional antibodies.

## Materials and methods

### Cell lines, culture and preparations

The HBME line HBMEC-60 was kindly provided by Dr. C. E. van der Schoot, University of Amsterdam (Amsterdam, The Netherlands). The HBMEC-60 cells were immortalized using the amphotrophic helper-free retrovirus pLXSN16 E6/E7 and have shown to maintain their normal phenotype and adhesive properties, specifically their ability to bind hematopoietic progenitor cells [36]. Basal Medium 200 (Invitrogen) supplemented with 20% fetal bovine serum (FBS, Atlanta Biologicals, Lawrenceville, GA, USA), gentamicin (Invitrogen) and low-serum growth supplement (Invitrogen) containing hydrocortisone, human fibroblast growth factor, heparin, and human epidermal growth factor was utilized for growing HBMEC-60. Highly metastatic human breast cancer cell lines, MDA-MB-231 (MB231) purchased from American Type Culture Collection (ATCC, Manassas, VA, USA), and MDA-MB-435 (MB435) kindly provided by Dr. J. Price (M.D. Anderson Cancer Center, Houston, TX), were used in this study. Both tumor cell lines were routinely cultured in RPMI 1640 medium (Invitrogen) supplemented with 10% FBS, L-glutamine and gentamicin. All cells were maintained as monolayer cultures in a humidified incubator (Heraeus Instruments, Newtown, CT) in 5% CO_2_ at 37°C.

For adhesion experiments, endothelial cells were plated in collagen-I coated Petri dishes (BD Bioscience) containing endothelial culture media. Cells were generally grown for 2 to 3 days to achieve confluence. In order to avoid possible variance introduced by cell passage and culture period, cells were used at a similar range of passages and seeded at initial densities producing the desired degree of confluence within 2–3 days. To avoid over-crowding, endothelial cells were used for AFM experiments within 16 hours of reaching a confluent monolayer. Cells were washed once and the growing media was replaced with serum-free, CO_2_ independent medium (CO_2_IM, Invitrogen) before an experiment.

MB231 or MB435 cells at 40–50% confluence were detached in Cell Dissociation Buffer (Invitrogen) before an experiment. After centrifugation, cells were re-suspended in CO_2_IM and used for AFM cantilever attachment. All cancer and endothelial cells were allowed to equilibrate for 15 min in CO_2_IM in the atmosphere at room temperature prior to AFM experiments, and continuously maintained in the same conditions during the experiments.

### Antibodies and other reagents

The following functional antibodies were used in this study: anti-TF-Ag antibody produced by JAA-F11 hybridoma [37]; mouse monoclonal anti-human integrin α3 (α3, clone P1B5, Millipore); anti-human integrin β1 (β1, HUTS-21, BD Pharmingen); anti-human integrin β1 activating (activ β1, P4G11, Millipore); and rat anti-human/mouse Gal-3 (M3/38, Santa Cruz Biotech). Other reagents used in the study are: biotinamidocaproyl labeled BSA (Sigma-Aldrich); Streptavidin (Sigma-Aldrich); Biotinylated concanavalin A (Con A, Sigma-Aldrich); and 16% paraformaldehyde (Electron Microscopy Sciences).

### Atomic force microscopy

All AFM experiments including measurements of cell-cell adhesion were performed using an Asylum Research AFM System (Model MFP-3D-BIO, Asylum Research, Santa Barbara, CA) with IGOR Pro software (WaveMetrics Inc., Oregon). The system was mounted on an inverted optical microscope (Model IX81, Olympus America Inc.).

### Cantilever functionalization and cell attachment

Tipless nitride cantilevers (MLCT-O10, Bruker Corp.) with a nominal spring constant of 30 pN/nm were used for measurements of cell adhesion force. Cantilevers were calibrated after a given experiment using the thermal noise method [38]. The measured spring constants were typically found to be between 8 and 14 pN/nm. The cantilever functionalization with Con A was performed following procedures that were previously described by Zhang et al. [39]. Briefly, the cantilevers were treated in 0.5 mg/ml biotin labeled BSA overnight, then washed three times in PBS followed by incubation in 0.5 mg/ml streptavidin. The cantilevers were finally incubated in 0.5 mg/ml biotinylated Con A at room temperature and rinsed in PBS. The functionalized probe was mounted on the AFM head and placed in a culture dish that was on the microscope stage and contained suspended cancer cells in CO_2_IM. Cancer cell to cantilever attachment was performed manually under the optical microscope. Single cancer cells of similar size were selected in order to keep the cell contact area as consistent as possible throughout experiments. The tip of the Con A-coated cantilever was aligned with the center of the cell and gently lowered onto the cell for 1 sec. The cancer cell was lifted by retraction of the cantilever and allowed to rest for 5 min to allow the cell to adhere firmly to the cantilever.

### Cell-cell adhesion measurement

**Fig 1:**
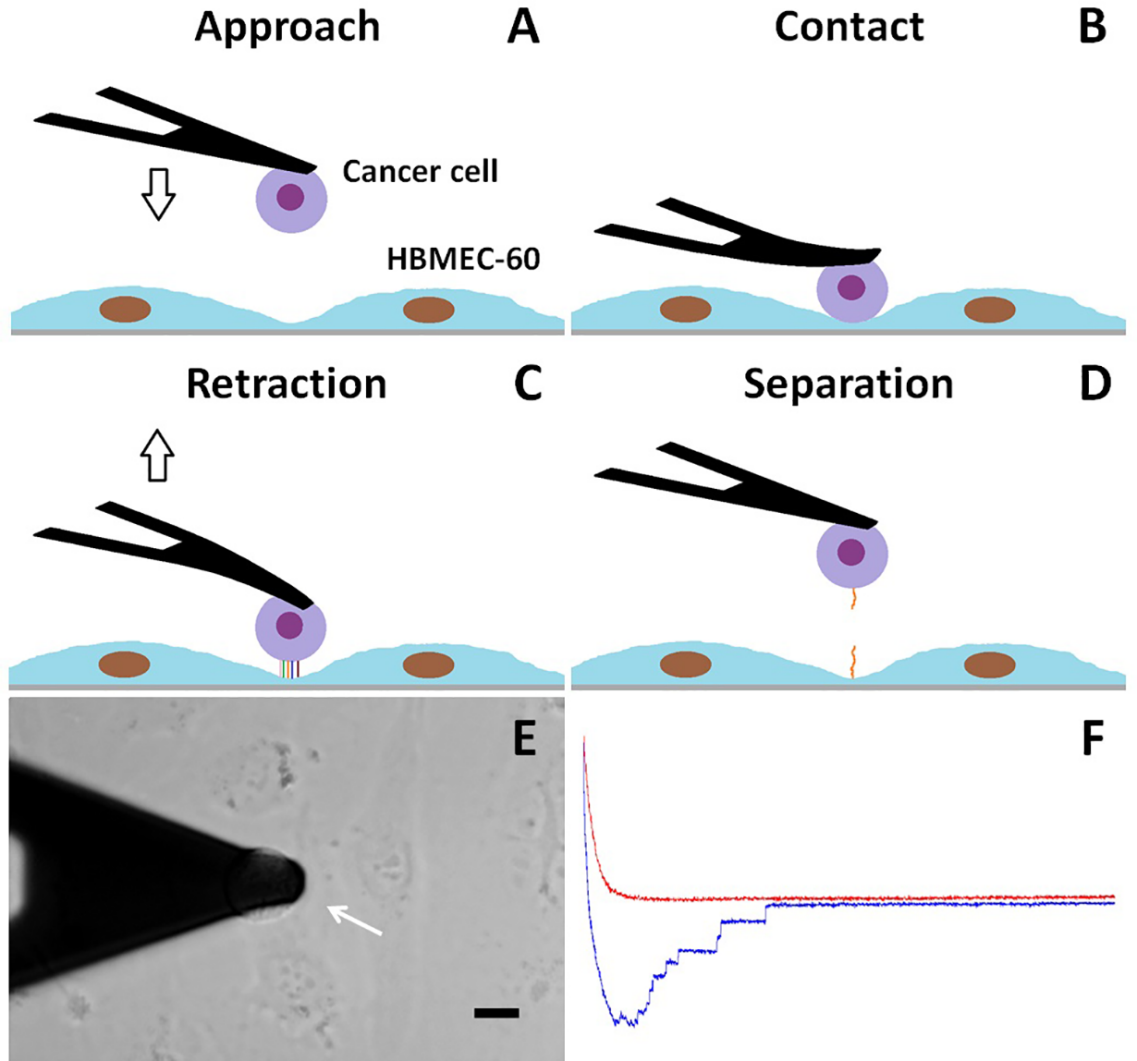
Diagrams and representative pictures of cancer-endothelial cell adhesion measurement. A single cancer cell is attached on the end of the Con A-coated tipless cantilever (A) and then brought into contact with the endothelial (HBMEC-60) cell-cell junctions (B). After a defined contact time, the cantilever is retracted (C) until the cancer cell is entirely separated from endothelial cells (D). The phase contrast image (E) shows an MB435 cell (white arrow) attached to the cantilever and in contact with a HBMEC-60 cell-cell junction. Force curves (F) are acquired during the cantilever approach (red) and retraction (blue), and the retraction curve is used to calculate adhesion forces. Scale bar in E = 10 µm.

For detecting adhesive forces between cancer cells and the HBME monolayer, the cantilever with a coupled cancer cell was introduced into a Petri dish containing the HBMEC-60 at confluence. The attached tumor cell was positioned to come into contact with an endothelial cell-cell junction (Fig 1). The endothelial junction was chosen to measure because these regions are the putative sites of cell extravasation, and maximal adhesion forces have been reported for these sites [14]. The range of cantilever force distance approach-retraction was adjusted to at least 40 μm to make it possible to completely separate the tumor cell from the endothelial cells when the cantilever retracts. The closed loop feedback mode of the piezoelectric position was applied in the AFM system to minimize the vertical drift of the cellular probe [33]. The closed loop feedback mode of the piezoelectric position was default enabled in the AFM system in order to keep constant the position of the cell-cell contact. The sampling frequency was set at 0.02 Hz, resulting in a cantilever velocity of 1.60 μm/s. The average cell-cell contact force was 522 pN. The contact-time dependence of cell-cell adhesion forces were measured by running cantilever approach-retraction cycles that brought the cancer cell into contact with endothelial cells for different cell-cell contact periods (0.5–300 sec) prior to cantilever retraction. Fifteen curves were collected for each contact time point on one sample. For negative adhesion control, the cancer cell was brought into contact with the bottom of a hydrogel coated dish (Corning Incorporated, NY).

For adhesion experiments in samples treated with functional antibodies, baseline adhesion forces between cancer and HBME cells were first obtained for cell-cell contact times of 0.5, 30, and 60 sec. An antibody was then added into the dish and both cancer and HBME cells were incubated with the antibody for 40 min at room temperature. Force experiments were repeated in the presence of the antibody. One cancer cell used for adhesion measurements was defined as one sample number (n).

Adhesion forces were analyzed from retraction curves with MATLAB and calculated in pN. The force that is required to break a single ligand-receptor bond was defined as an individual adhesive interaction (recorded as a rupture force) and the total force that was necessary to completely separate the cancer cell from HBMEC-60 monolayer was defined as the aggregate total (overall) force to rupture all adhesions (total adhesion force, adhesion strength). The term “rupture” is used to define an abrupt decrease of deflection in the force curve. The retraction curve can contain a number of rupture events before the deflection returns to the original level (i.e. when cantilever is free of mechanical loads). To isolate and calculate individual rupture events, a protocol with three steps of filtering was incorporated in the data analysis programming. The first threshold (1 nm) was set to filter the rupture by the size of change in deflection and the second threshold (0.28) was set to filter the rupture by the slope of change in deflection. If the rupture event passed both filters, the force curve sections before and after the deflection change were linearly fitted, and the difference of deflection between these two sections was tested against the third threshold (1 nm). Only rupture events that pass all three filtering steps were recorded and calculated. The method was validated by comparing with human eye to assure more than 95% rate of capturing of ruptures that could be detected by human eye. The mean total adhesion forces for single cancer cells were calculated and further averaged together for each group. For data from the cancer-HBME adhesion study with antibody treatment, a percentage of adhesion changes was calculated when the adhesion force value from an antibody treated cell was divided by its own baseline. The distributions of rupture forces were plotted using Origin ver. 8.6 software.

### Statistical analysis

All data are expressed as a mean ± standard error of the mean (SEM). Percentage data from cancer-HBME adhesion studies with treatments of functional antibodies were compared using a paired t-test. Multiple comparisons for studies of the dependence of adhesion on contact-time between the cancer cells and the endothelial cells were analyzed with ANOVA. A value of P< 0.05 was considered significant for all comparisons.

## Results

### Cancer-HBME adhesion with cell-cell contact duration

**Fig 2:**
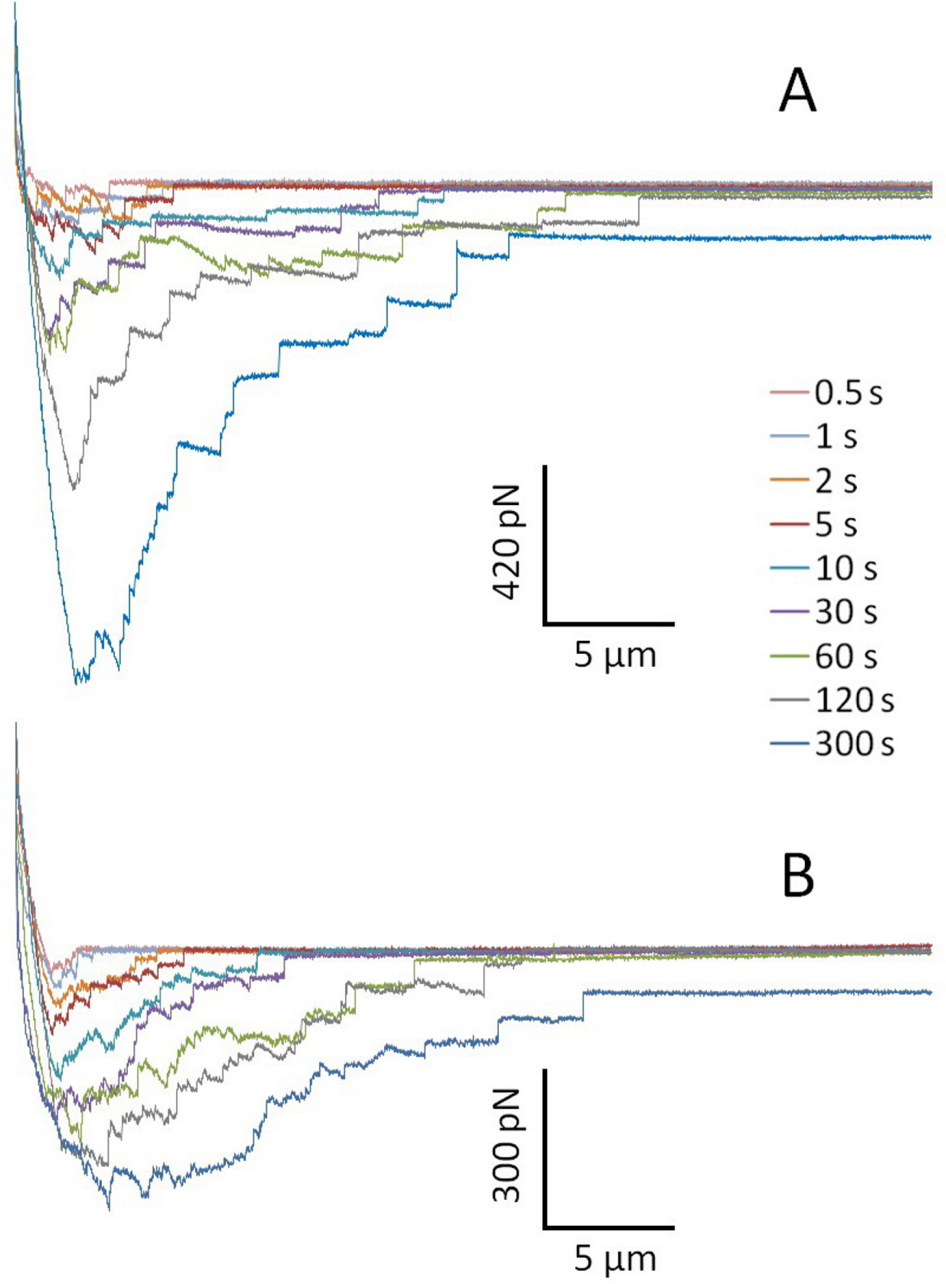
Typical adhesion force curves of MB435 (A) and MB231 (B) to HBMEC-60 as a function of increasing contact time. Representative curves for AFM cantilever retraction that were recorded in the experiments were plotted for increasing lengths of cell-cell contact time in seconds (s). The total force required to separate the tumor cell from the endothelial cells increased as a function of increasing contact time. The peak force in the aggregate curve for both MB435 (A) and MB231 (B) cells shifts temporally to the right with increasing duration of contact. The vertical and horizontal black bars stand for adhesion force (pN) and cantilever retraction distance (µm), respectively.

**Fig 3:**
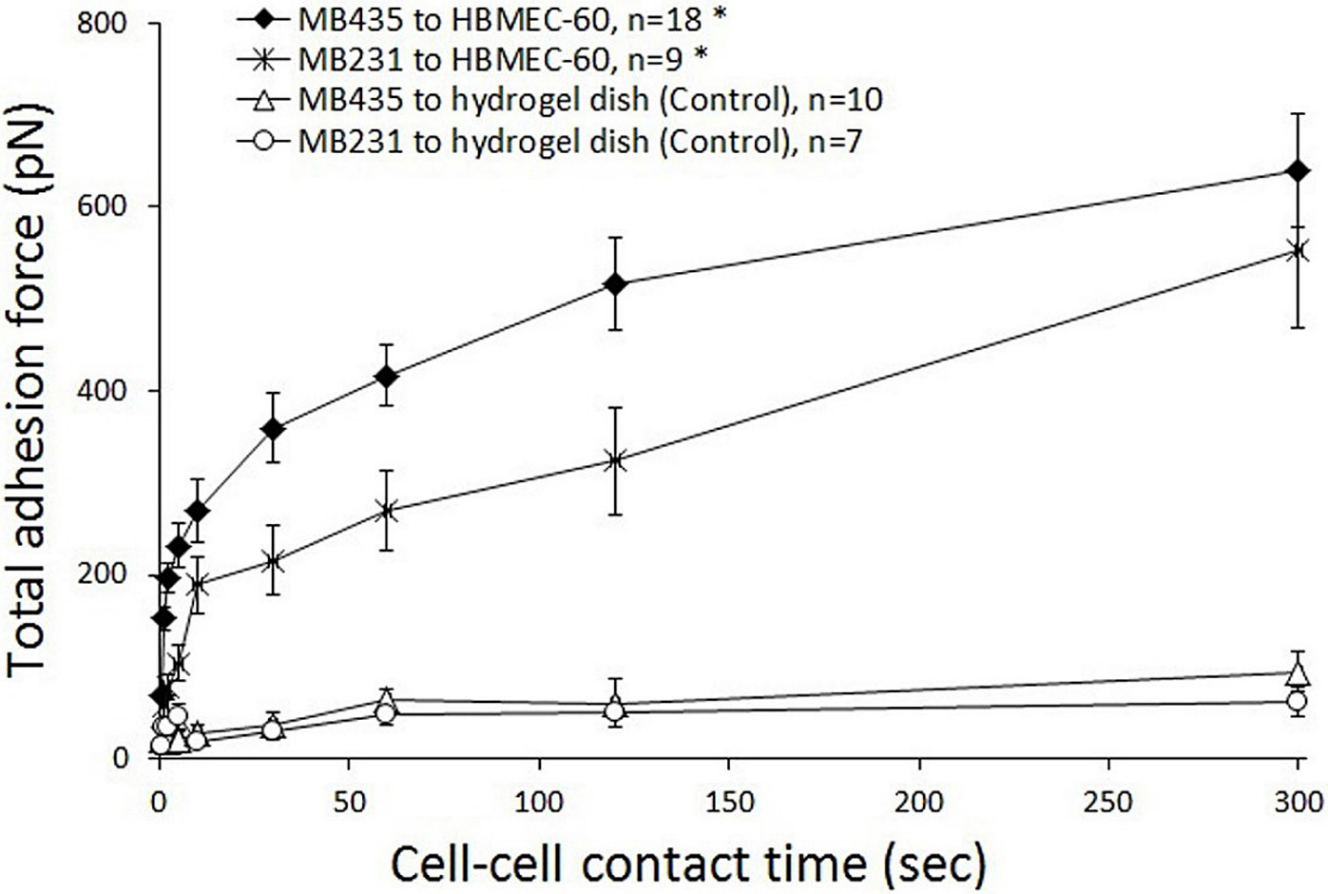
A relationship between the total cancer-HBME adhesion and duration of cell-cell contact. Adhesion strength between MB231 and MB435 to HBMEC-60 cells was measured for different contact times: 0.5, 1, 2, 5, 10, 30, 60, 120, and 300 sec. The overall total adhesion forces in MB231 (asterisks) cells at all time points were lower than those in MB435 (diamonds). In both cell lines, from 0.5 to 300 sec cell-cell contact time the total adhesion forces increased progressively 23.79-fold (from 23.27 ± 5.73 to 553.64 ± 86.10 pN) and 9.29-fold (68.81 ± 5.91 to 639.37 ± 62.50 pN) for MB231 and MB435 respectively. Note similar time courses of adhesion dynamics to HBMEC-60 displayed by both cancer cell lines, whereas the adhesion force increased dramatically within the first 10 sec (MB231) or 30 sec (MB435) of contact with HBMEC, and then more gradually As a negative control, the MB435 (triangles) and MB231(circles) cells were brought into contact with a hydrogel-coated dish instead of an endothelial monolayer. *p< 0.001 by ANOVA analysis.

By coupling the cancer cell to the end of AFM cantilever and bringing it into contact with cell-cell junctions of the HBME monolayer for various cell-cell contact periods (0.5, 1, 2, 5, 10, 30, 60, 120 and 300 sec), adhesions between individual cancer cells and HBME cells were quantified to observe the correlation between the cancer-endothelial cell adhesion and length of cell contact time. Plotted in Fig 2 are typical AFM cantilever retraction curves that were recorded in adhesion studies of MB435/MB231 to HBMEC-60 cells with various cell-cell contact times. The increases in peak force and total separation force (area between the approach and retraction curves, see Fig 1F) with times for both cancer cell lines are similar, clearly indicating that the force required for separation of the tumor cell from the endothelial cells increased as a function of increasing contact time. It is seen that peak forces for both MB435 and MB231 in the aggregate curve shift to the right with increasing duration of contact, indicating that a transiently stretched deformation of cancer and/or endothelial cells occurred upon them separating at the initial retraction and suggesting adaptive cytoskeletal rearrangements to support adhesion as well as engagement of more adhesion molecules. Correlations between cancer-HBME adhesion strength and the duration of cell-cell contact are detailed in Fig 3. Although the total adhesion forces in MB231 cells at all time points were lower than those in MB435, adhesion forces increased progressively up to 23.79-fold (from 23.27 ± 5.73 to 553.64 ± 86.10 pN) for MB231 when cell-cell contact time was varied from 0.5 to 300 sec, while 9.29-fold (68.81 ± 5.91 to 639.37 ± 62.50 pN) for MB435. Moreover, the two cancer cell lines displayed similar time courses of adhesion to HBMEC-60, in which the adhesion strength in MB231 increased dramatically within the first 10 sec of contact and in MB435 within the first 30 sec, and then more gradually. Force data at increasing cell-cell contact times for both MB231 and MB435 cells were extremely significant (p< 0.001) by single factor ANOVA. As a negative control, the cancer cells were brought into contact with a hydrogel-coated dish instead of an endothelial monolayer (Fig 3). We found that total adhesion forces between cancer cells and the hydrogel-coated dish were very low and did not increase when cell-dish contact time was extended even for the longest contact period (300 sec). These results demonstrate that the adhesion strength of MB231 and MB435 to HBMEC-60 cells is strongly dependent on cell-cell contact time, especially within the initial 10 or 30 sec.

### Rupture forces in the contact-time dependent adhesion

**Fig 4:**
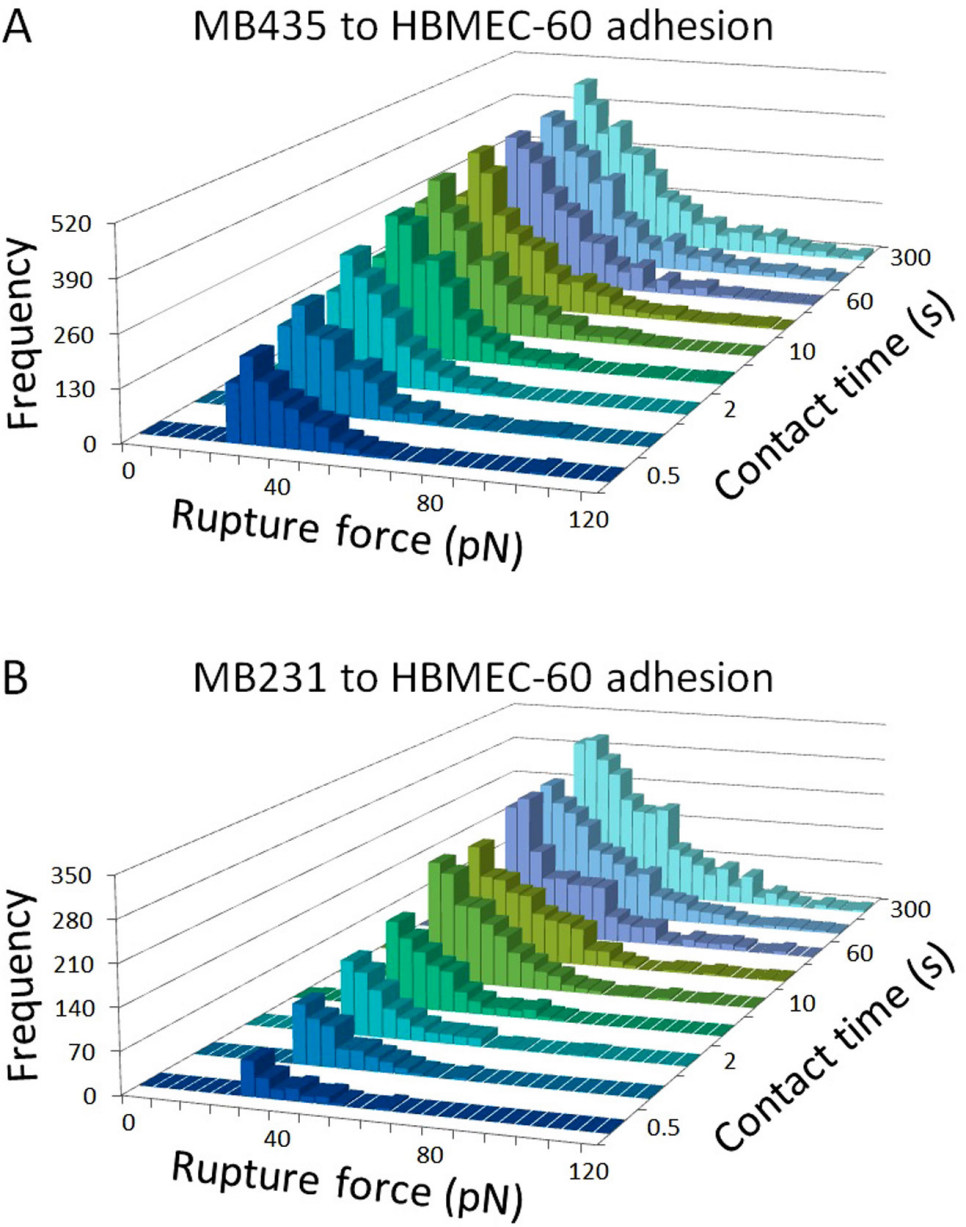
Distributions of rupture forces detected during cancer-HBME adhesion with cell-cell contact times of 0.5, 1, 2, 5, 10, 30, 60, 120 and 300 sec. Note 3.89-fold increase (from 903 to 3516 counts) in the frequency counts of the detectable rupture events for MB435 and 15.39-fold increase (from 156 to 2401 counts) for MB231 from 0.5 to 300 sec of cell-cell contact. Also note a significant shift of the rupture force frequency distribution histograms to the right for both cancer cell lines. Similar changes in histograms of rupture force distribution for both MB435 (A) and MB231 (B) cells indicate that both the increase in frequencies of individual adhesion events and involvement of stronger ligand-receptor interactions contributed to the change in total adhesion force over time.

To assess how the strength of individual ligand-receptor binding involved in cancer-HBME adhesion changed with extended cell contact time, we analyzed individual rupture forces and compared the magnitude and frequency of occurrence of the forces (Fig 4). The frequency counts of detectable rupture events increased 3.89-fold (from 903 to 3516 counts) in MB435 and 15.39-fold (from 156 to 2401 counts) in MB231 from 0.5 to 300 sec of cell-cell contacts. Changes in the force distribution histograms were similar in shape for both MB435 (Fig 4A) and MB231 (Fig 4B) cells showing marked peak distribution shifting to the right as contact time increased, which is indicative of the involvement of stronger ligand-receptor-cytoskeletal adhesive interactions. Although, the possibility that simultaneous rupture of several weaker ligand-receptor bonds could be reflected as a single abrupt decrease of deflection on the force curve (and detected as a single rupture of a stronger adhesive bond) can never be excluded, for the purpose of this study, each such event was accounted as an individual rupture. Collectively, these data indicate that changes in both the number of adhesions and in magnitude of individual adhesion rupture forces are responsible for the contact time dependence of total cancer-HBME adhesion forces.

### Molecule involvements in cancer-HBME interactions

**Fig 5:**
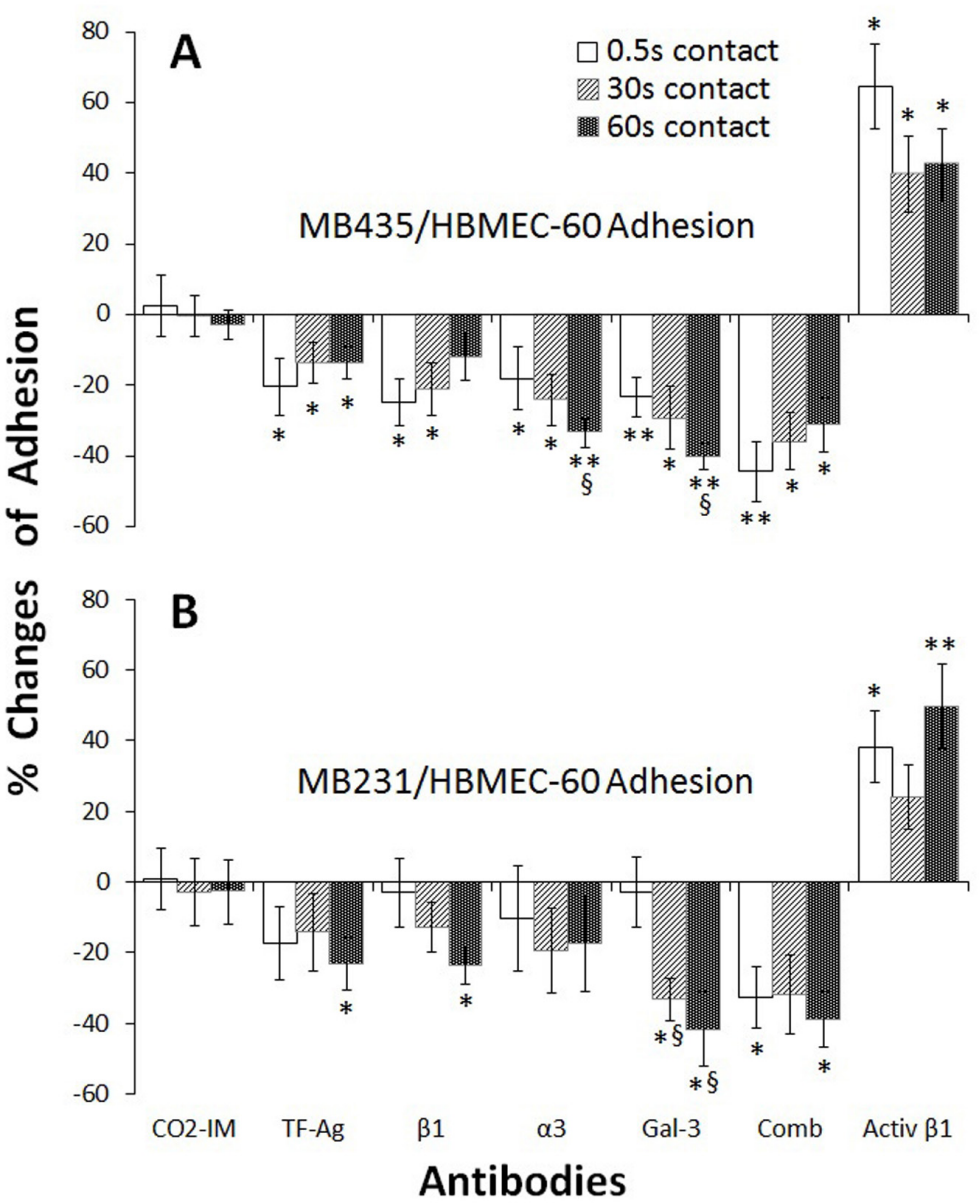
Changes in cancer-HBME total adhesion force with antibody treatment. In adhesion between MB435 and HBMEC-60 (A), from 0.5 to 30 to 60 sec contact times, total adhesion forces were significantly reduced by antibodies to TF-Ag and β1 showing descending inhibition dynamics with the highest inhibition at 0.5 sec, compared to the baseline; while anti-α3 and anti-Gal-3 showed ascending inhibition dynamics with the highest inhibition at 60 sec. As positive control, activating β1 antibody (Activ β1) induced the greatest adhesion (64.55%) at 0.5 sec, which is in agreement with what was observed with inhibiting anti-β1 antibody. In adhesion between MB231 and HBMEC-60 (B), significant inhibition was observed at the 60 sec contact time with antibodies against TF-Ag (-23.26%), β1 (-23.81%) and Gal-3 (-41.71%), but not statistically significant with anti-α3, compared to the baseline. *p< 0.05; **p< 0.01, vs the baseline. § p< 0.05 vs 0.5 sec contact. n> 6.

In order to identify different types of molecules supporting cancer-HBME adhesive interactions and to observe whether the molecular involvements are time-dependent, we performed pre-incubations of both cancer and HBME cells with function-blocking antibodies (10 µg/ml) against TF-Ag, integrin β1, integrin α3, and galectin-3 (Gal-3) for 45 min. Next, adhesion forces were measured for cell-cell contact times of 0.5, 30 and 60 sec before (baseline) and after treatment with the antibodies. CO_2_IM without antibody was used as a negative control, while an activating anti-integrin β1 (activ-β1) antibody was used as a positive control. Changes in adhesion forces were calculated and illustrated in Fig 5. Upon interaction between MB435 and HBMEC-60, adhesions were significantly reduced to the various degrees by function-blocking antibodies (Fig 5A). From contacts of 0.5 to 60 sec, the highest inhibition at 0.5 sec was seen with antibodies to TF-Ag (-20.45 ± 7.92%, n = 10, p< 0.05) and β1 (-24.89 ± 6.56%, n = 14, p< 0.05), which had descending inhibitions with increase of contact, compared to the baseline; while α3 (-33.41 ± 4.18%, n = 11, p< 0.01) and Gal-3 (-40.27 ± 3.68%, n = 10, p< 0.01) with the highest inhibitions at 60 sec, showing ascending inhibitions with prolonged contact, when compared to the baseline. In the positive control, activ-β1 antibody induced the greatest adhesion (64.55 ± 12.05%, n = 10, p< 0.05) at 0.5 sec in the three time points, which is in agreement with what was seen in the group treated with inhibiting β1 antibody. There are significant time-dependencies at the contact time of 60 sec in α3 and Gal-3 antibody treated groups, compared to the 0.5 sec (p< 0.05).

In adhesion between MB231 and HBMEC-60 (Fig 5B), antibodies against TF-Ag (-23.26 ± 7.44%, n = 10, p< 0.05) and β1 (-23.81 ± 5.07%, n = 8, p< 0.05) revealed inhibitions in the adhesion for 60 sec of the contact, but not for 0.5 and 30 sec. Antibody to Gal-3 inhibited significantly adhesion for 30 sec (-33.40 ± 5.89%, n = 7, p< 0.05) and 60 sec (-41.71 ± 10.51% for 60 sec, n = 7, p< 0.05) contact time, when compared to the baseline; and Gal-3 antibody had significantly time-dependent inhibitions for 30- and 60-sec contact time (p< 0.05), compared to the 0.5 sec (-2.95 ± 9.94%, n = 7). Inhibition of MB231 adhesion by TF-Ag and β1 antibodies for 0.5 sec contact time, as well as by α3 antibody for all three time points did not reach statistical significance due to the insufficient sample size.

**Fig 6:**
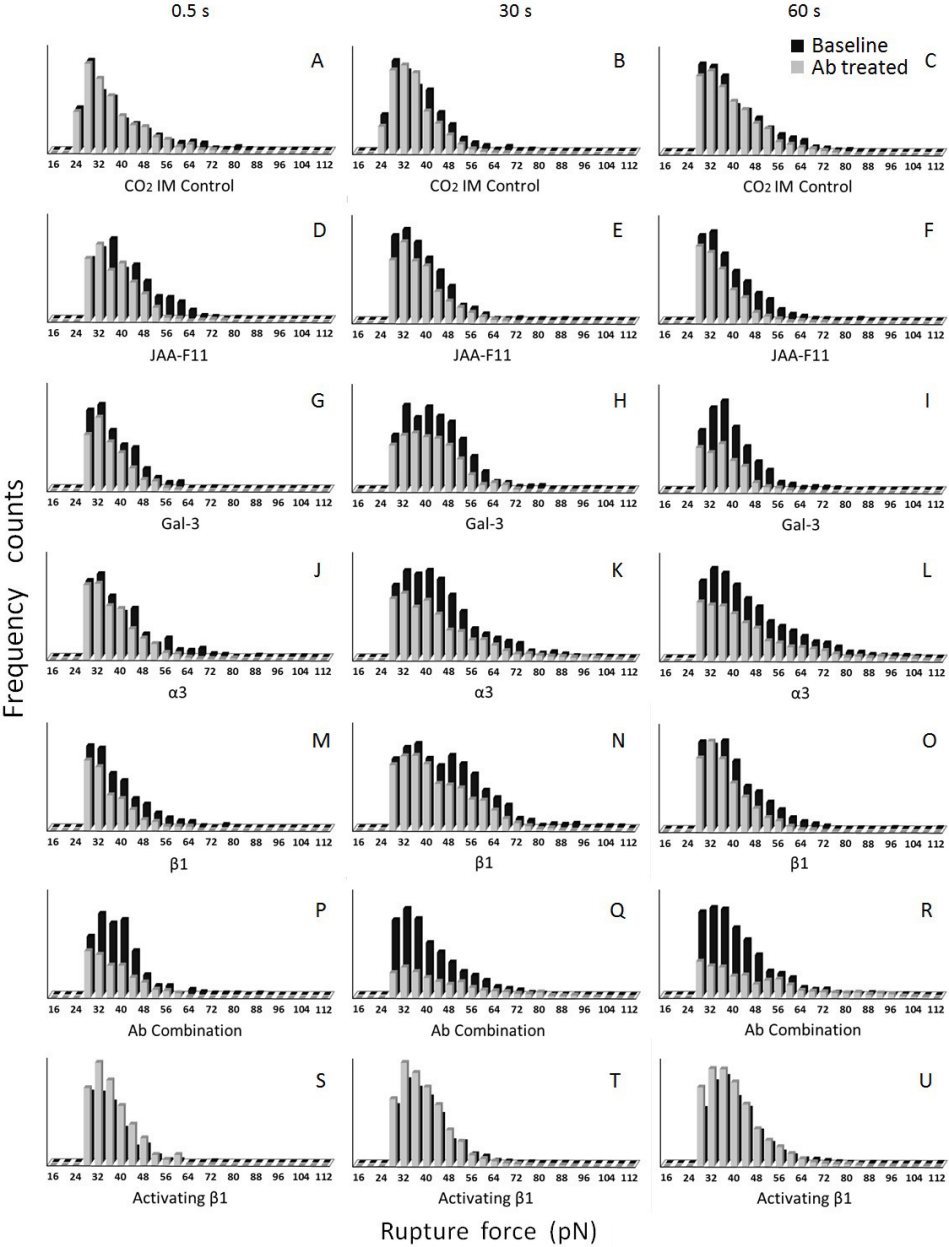
Distribution frequencies of rupture forces collected from MB435-HBMEC-60 adhesion for cell-cell contacts of 0.5 (A,D,G,J,M,P, and S), 30 (B,E,H,K,N,Q, and T) and 60 (C,F,I,L,O,R, and U) seconds before and after antibody (10 µg/ml) treatments. There were no changes seen in rupture distribution in CO_2_IM treated control (A, B and C). Function-inhibiting antibodies to TF-Ag (D, E and F), Gal-3 (G, H and I), α3 (J, K and L) and β1 (M, N and O) decreased the number of rupture events compared to the respective baselines. Note reduced number of the weakest (30–50 pN) and midrange (50–80 pN) adhesions at the earliest 0.5 sec time point caused by anti-TF-Ag (D), which apparently prevented the formation of stronger adhesions at 30 and 60 sec time points (E and F). Anti-Gal-3 antibody caused rather limited inhibitory effect on the weakest (30–50 pN) and midrange (50–80 pN) adhesions at 0.5 sec time point (G) It demonstrated, however, a pronounced inhibition of adhesions across the entire spectrum at 30 and 60 sec (H and I), indicating that Gal-3 involvement requires additional time to unfold, and that stronger, integrin-mediated adhesive events, may depend on preceding Gal-3 mobilization. Limited inhibitory effect was detected with function-blocking antibodies against integrins α3 and β1 at 0.5 sec (J and M respectively). At 30 and 60 sec time points, however, both anti-α3 (K and L) and anti-β1 (N and O) caused more significant inhibition of adhesions across the entire spectrum including the stronger (>80 pN) adhesions. A combination of the four function-blocking antibodies (P-R) showed a greater inhibitory effect than any of the antibodies alone (D to O). In the positive control (S-U), the function-activating antibody to β1 increased the number of detectable ruptures.

Analyses of rupture forces collected from cancer-HBME adhesion measurements demonstrated that treatments with inhibitory antibodies reduced the number of observed rupture forces and changed the shape of the frequency distribution as well. Plotted in Fig 6 are distribution histograms created from MB435-HBMEC-60 adhesion for cell-cell contacts of 0.5 sec (Fig 6A, 6D, 6G, 6J, 6M, 6P and 6S), 30 sec (Fig 6B, 6E, 6H, 6K, 6N, 6Q and 6T), and 60 sec (Fig 6C, 6F, 6I, 6L, 6O, 6R and 6U) before and after antibody (10 µg/ml) treatment. There were no obvious changes seen in rupture distribution in CO_2_IM treated control (Fig 6A–6C). Function-inhibiting antibody to TF-Ag (Fig 6D–6F), noticeably reduced the number of the weakest (30–50 pN) and midrange (50–80 pN) adhesions at the earliest 0.5 sec time point (Fig 6D), which apparently prevented the formation of stronger adhesions at 30 and 60 sec time points (Fig 6E and 6F). Anti-Gal-3 antibody had some inhibitory effect on midrange (50–80 pN) adhesions at 0.5 sec time point (Fig 6G), but a very pronounced inhibition of adhesions across the entire spectrum at 30 and 60 sec (Fig 6H and 6I), indicating that Gal-3 involvement may require additional time to unfold, and that stronger, integrin-mediated adhesive events, may depend on preceding Gal-3 mobilization. Function-blocking antibodies against integrins α3 (Fig 6J–6L) and β1 (Fig 6M–6O) had limited inhibitory effect at 0.5 sec (although more pronounced for β1 suggesting that other β1 integrins in addition to α3β1 could be involved). At 30 and 60 sec time points, however, both anti-α3 and anti-β1 caused more significant inhibition of adhesions across the entire spectrum including the stronger (>80 pN) adhesions. As expected, a mixture of all four function blocking antibodies (Fig 6P–6R) caused substantially more significant inhibition of adhesions across the entire spectrum than any of the antibodies did alone.

**Fig 7:**
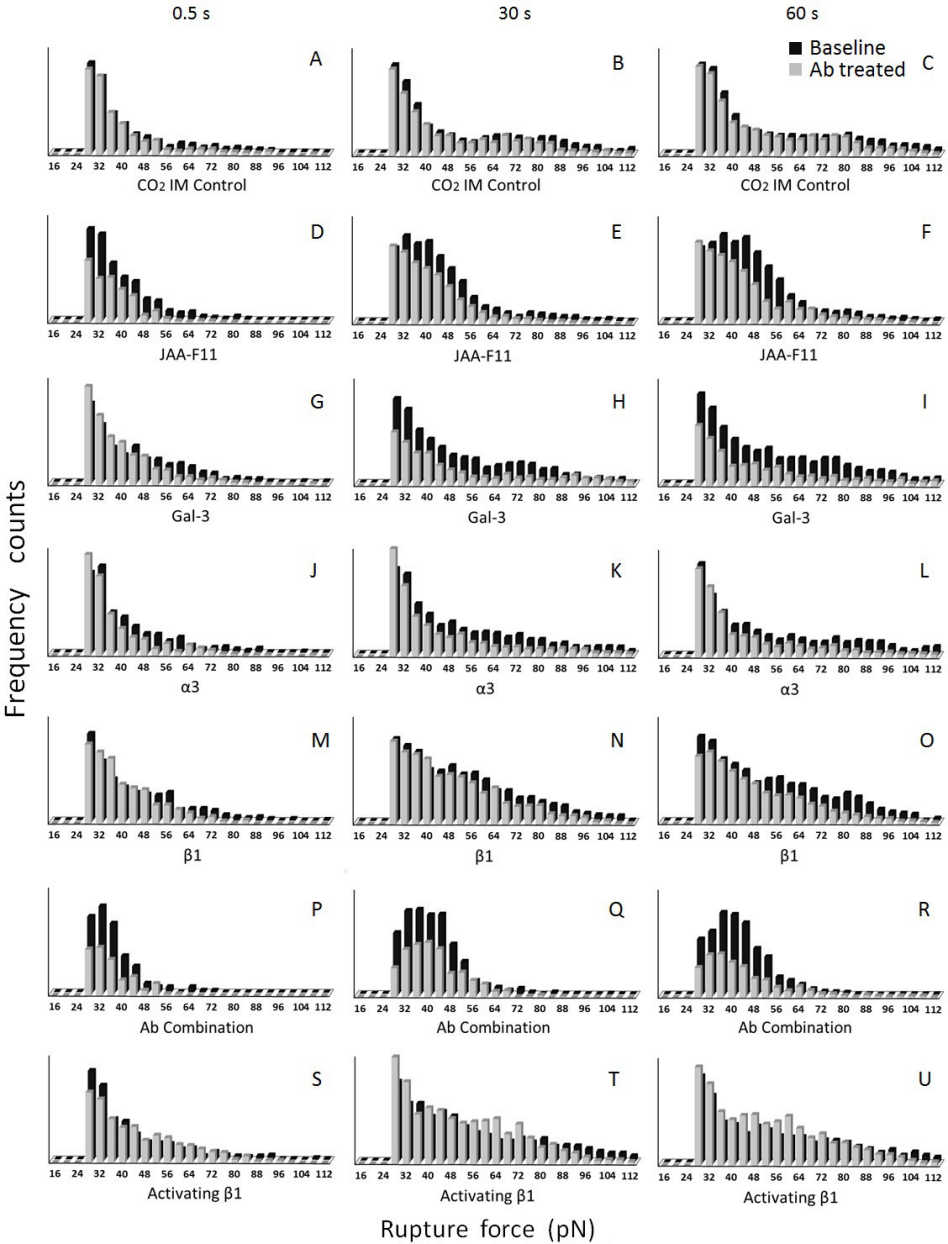
Distribution frequencies of rupture forces collected from MB231-HBMEC-60 adhesion experiments for cell-cell contact times of 0.5 (A,D,G,J,M,P, and S), 30 (B,E,H,K,N,Q, and T) and 60 (C,F,I,L,O,R, and U) sec before and after antibody (10 µg/ml) treatment. No obvious changes were detected in rupture distribution in CO2IM treated controls (A-C) compared to the baseline. Noticeable reduction in the number of the weakest (30–50 pN) and midrange (50–80 pN) adhesions at the earliest (0.5 sec) time point was caused by anti-TF-Ag JAA-F11 antibody (D), which also prevented the formation of stronger adhesions at 30 and 60 sec time points (E and F). Only minimal inhibitory effect on midrange (50–80 pN) adhesions was detected at the earliest (0.5 sec) time point with anti-Gal-3 antibody (G). However, this same antibody caused very pronounced inhibition across the entire spectrum of adhesions at 30 and 60 sec (H and I). The inhibitory effect of function blocking anti-α3 and anti-β1 antibodies was rather limited at 0.5 sec time point (J and M respectively). They did cause, however, noticeable inhibition of adhesions across the entire spectrum at 30 and 60 sec time points (K, L, N and O). The inhibitory effect of the mixture of all four antibodies was significantly more pronounced across the entire spectrum of adhesions at all three time points (P-R) than any of the function blocking antibodies alone. In the positive control (S-U), the function-activating antibody to β1 increased the number of detectable ruptures.

Similarly, in MB231-HBMEC-60 adhesion experiments, there were no obvious changes in rupture distribution in CO_2_IM treated control experiments (Fig 7A–7C). Anti-TF-Ag JAA-F11 antibody (Fig 7D–7F), caused noticeable reduction in the number of the weakest (30–50 pN) and midrange (50–80 pN) adhesions at the earliest (0.5 sec) time point (Fig 7D), which prevented the formation of stronger adhesions at 30 and 60 sec time points (Fig 7E and 7F). As with MB435, anti-Gal-3 antibody caused some inhibitory effect on midrange (50–80 pN) adhesions at 0.5 sec time point (Fig 7G), and very pronounced inhibition of adhesions across the entire spectrum at 30 and 60 sec (Fig 7H and 7I). The dynamics of inhibition of MB231 cells by function-blocking antibodies against integrins α3 (Fig 7J–7L) and β1 (Fig 7M–7O) were also similar to those of MB435. That is, they both had rather limited inhibitory effect at 0.5 sec (Fig 7J and 7M), but caused more significant inhibition of adhesions across the entire spectrum at 30 and 60 sec time points (Fig 7K, 7L, 7N and 7O). Again, as with MB435, the mixture of all four antibodies caused significantly more pronounced inhibition across the entire spectrum of adhesions at all 3 time points (Fig 7P–7R).

Collectively, these results are in agreement with our previously proposed model whereby tumor/endothelial cell adhesion is initiated by TF-Ag interactions with endothelial Gal-3 causing translocation and clustering of the later at the endothelial cell membrane and followed by the mobilization of α3β1 integrin further strengthening and stabilizing these adhesive interactions [13].

## Discussion

Metastatic cell interactions with vascular endothelium in distant organs constitute an essential component of hematogenous cancer metastasis. Tumor/endothelial cell adhesion is not simply an act of the mechanical attachment of circulating blood-borne metastatic cells to the vessel wall, but an extremely complex and dynamic process. This process is tightly regulated on many levels [13] and involves highly orchestrated interactions of multiple ligand-receptor pairs, cytoskeleton rearrangements, and major signaling pathways. In this study, we have used AFM to investigate temporal dynamics of the adhesion of two human metastatic breast carcinoma cell lines, MDA-MB-231 and MDA-MB-435, to human bone marrow endothelial cells HBMEC-60. Not only did use of AFM provided for a quantitative characterization of cell-to-cell adhesive interactions with unprecedented (piconewton) resolution, but also it afforded the possibility for studying the process of maturation of tumor/endothelial cell adhesion via the engagement of new, quantitatively and qualitatively distinct molecular events, as well as interrogating the involvement of individual ligand-receptor pairs in the process.

Our results indicate that the strength of adhesion between endothelial cells and both MB435 and MB231 cells increases dramatically within first 10–30 sec and then more gradually, resulting in the total increase in adhesion strength for MB435 and MB231 cells 23.79-fold and 9.29-fold respectively within 300 sec (Fig 3). Evidently, this change occurs due to both the increase in the number of individual adhesion events (3.89-fold for MB435 and 15.39-fold for MB231), and involvement of qualitatively different (stronger) adhesions (Fig 4). These results indicate that after the initial attachment of tumor cell to the endothelial cell via weaker adhesive interactions, a rapid process of tumor-endothelial cell adhesion maturation and stabilization takes place involving exponential increase in the number of adhesion molecules engaged and development of much stronger individual adhesive interactions. This process can involve translocation toward and clustering at the sites of adhesion the types of molecules already engaged into tumor-endothelial cell interactions from the very beginning (for example, TF-Ag on tumor cells and endothelial Gal-3) resulting in the multiplication of the adhesions and strengthening of the overall bond between tumor and endothelial cells. It is likely as well to involve the recruitment of new ligand-receptor pairs including molecules with stronger adhesive properties such as integrins.

Recently, based mostly on prostate cancer experiments, we have proposed the model, whereby adhesive interactions between tumor and endothelial cells initiated by cancer-associated TF-Ag causes translocation and clustering of the endothelium-expressed Gal-3 at the sites of adhesion, which in turn signals mobilization of the α3β1 integrin that strengthens and stabilizes tumor/endothelial cell adhesion [13]. In this study, we have analyzed distribution frequencies of rupture forces collected from AFM experiments utilizing function blocking antibodies against TF-Ag, Gal-3, α3 and β1 integrin. Although the AFM experiments were performed on a different (much shorter) time scale, they have confirmed that these same molecules play important role in breast carcinoma cell adhesion to bone marrow endothelium as well. However, as none of these antibodies alone (or even all of them combined) were able to inhibit tumor/endothelial cell adhesion completely, it is likely that additional adhesion molecules are involved in the process. Further, based on the dynamics of the inhibition of tumor/endothelial cell adhesion by anti-α3 and anti-β1 antibodies in MB435 cell experiments it is apparent that in addition to α3β1 integrin, other β1 integrin molecules could be involved.

In summary, we have used AFM, a highly sensitive, robust method to characterize the temporal course of adhesion between human breast cancer cells and an endothelial monolayer and probe a number of molecules that are involved in the adhesive interaction between breast cancer cells and the endothelium. The AFM single-cell force spectroscopy provides unprecedented precision, resolution and sensitivity for interrogating the involvement of various adhesion molecules into the process of cancer metastasis.

## Supporting information

S1 File MB231-435-HBMEC60 adhesion curve overlapping. (XLSX)

S2 File MB231-435-HBMEC60 adhesion 0.5-300s combined. (XLSX)

S3 File Distribution MB231-435-HBMEC60 adhesion 0.5-300s. (XLSX)

S4 File MB435-HBMEC60 0.5s adhesion TFAg CD29 Ab 10ug 45m. (XLSX)

S5 File MB435-HBMEC60 30s adhesion TFAg CD29 Ab 10ug 45m. (XLSX)

S6 File MB435-HBMEC60 60s adhesion TFAg CD29 Ab 10ug 45m. (XLSX)

S7 File MB231-HBMEC60 0.5s adhesion TFAg CD29 Ab 10ug 45m. (XLSX)

S8 File MB231-HBMEC60 30s adhesion TFAg CD29 Ab 10ug 45m. (XLSX)

S9 File MB231-HBMEC60 60s adhesion TFAg CD29 Ab 10ug 45m. (XLSX)

S10 File Distribution MB231-435-HBMEC60 adhesion Ab. (XLSX)
